# Identification of transcriptionally active transposons in Barley

**DOI:** 10.1186/s12863-023-01170-1

**Published:** 2023-11-04

**Authors:** Dongying Gao, Emma Fox-Fogle

**Affiliations:** 1 Small Grains and Potato Germplasm Research Unit, USDA-ARS, Aberdeen, ID 83210 USA; 2 Present Address: National Agricultural Statistical Service, USDA, Olympia, WA 98501 USA

**Keywords:** Barley, Transposon, Expression, Genome, Comparative analysis

## Abstract

**Background:**

The genomes of many major crops including barley (*Hordeum vulgare*) consist of numerous transposons. Despite their important roles in crop genome evolution and morphological variations, most of these elements are silent or truncated and unable to be mobile in host genomes. Thus far, only a very limited number of active transposons were identified in plants.

**Results:**

We analyzed the barley full-length cDNA (FLcDNA) sequences and detected 71 unique FLcDNAs exhibiting significant sequence similarity to the extant transposase proteins. These FLcDNAs were then used to search against the genome of a malting barley cultivar ‘Morex’, seven new intact transposons were identified. Sequence alignments indicated that six intact transposons contained the entire FLcDNAs whereas another one served as 3’ untranslated region (3’ UTR) of a barley gene. Our reverse transcription-PCR (RT-PCR) experiment further confirmed the expression of these six transposons and revealed their differential expression. We conducted genome-wide transposon comparisons and detected polymorphisms of three transposon families between the genomes of ‘Morex’ and other three genotypes including the wild barley (*Hordeum spontaneum*, B1K-04-12) and two cultivated barley varieties, ‘Golden Promise’ and ‘Lasa Goumang’. Lastly, we screened the transcripts of all annotated barley genes and found that some transposons may serve as the coding regions (CDSs) or UTRs of barley genes.

**Conclusion:**

We identified six newly expressed transposons in the barley genome and revealed the recent mobility of three transposon families. Our efforts provide a valuable resource for understanding the effects of transposons on barley genome evolution and for developing novel molecular tools for barley genetic improvement and other research.

**Supplementary Information:**

The online version contains supplementary material available at 10.1186/s12863-023-01170-1.

## Background

Transposable elements (TEs) or transposons are genomic sequences that have the potential capacity to move within host genome and even horizontally transfer between distantly related organisms [1]. Thus far, transposons have been identified in the sequenced genomes of a wide range of organisms including microbes, plants, and animals [2]. They can be grouped into two major classes, Class I elements or retrotransposons mobilize via a copy-and-paste mechanism whereas Class II elements or DNA transposons transpose via the cut-and-paste or other mechanisms. Each major transposon class can be further divided into different superfamilies based on the sequence structures and encoded proteins such as retrotransposons are classified into long terminal repeat (LTR) retrotransposons and non-LTR retrotransposons including long interspersed nuclear elements (LINEs) and short interspersed nuclear elements (SINEs). Some transposon superfamilies are widely present in nearly all eukaryotes whereas other superfamilies show lineage-specific distributions and are only found in specific organisms. For example, among the 17 DNA transposon superfamilies, only 6 superfamilies are present in plant genomes [3].

The movements of transposons may cause deleterious or even lethal mutations that can affect host fitness [4, 5]. Therefore, the hosts and transposons have co-evolved multiple mechanisms to regulate transposon activity including DNA methylation, small interfering RNAs (siRNAs) and the genomic selection pressure [6–8]. Despite transposons contribute large fractions of many sequenced genomes, the majority of them were truncated or became dysfunctional due to the accumulations of mutations. Some transposons may be structurally intact, but their transcription is suppressed by the epigenomic silence pathways [6]. Thus far, active transposons were found to contribute tiny fractions of the eukaryotic genomes. For example, nearly half of the human genome is consisted of transposons, but only less than 40 subfamilies of Alu, L1 and SVA elements may still be active in the genome [9].

Active transposons are of great interest as they continue to create new insertions and genetic diversity that may have important impacts on structural and functional changes of genes and genomes [9, 10]. Additionally, identification of active transposons is helpful to understand the transposition mechanisms and develop molecular tools for cloning genes and identifying gene functions [11]. Some active transposons were originally found with molecular methods by comparing the functional genes between the wild types and mutants [12, 13]. With the availability of large amount of sequencing data, computational analysis has emerged as a popular strategy to identify potential active transposons by comparing the polymorphic insertions of transposons between related genomes or identifying highly identical transposon sequences within genome [10, 14]. Active transposons can be autonomous or nonautonomous elements, the former encode transposase protein(s) necessary for transposition, whereas the latter do not generate functional transposases and their movement is catalyzed by their autonomous partners [13, 14].

Barley (*Hordeum vulgare*, 2n = 2X = 14) is an important crop used for food, feed and malt worldwide. It belongs to the Triticeae tribe of the grass family which also contains bread wheat (*Triticum aestivum*, 2n = 6X = 42) and rye (*Secale cereale*, 2n = 2X = 14). Thus far, the genomes of both cultivated barley and its wild progenitor, *Hordeum spontaneum*, have been sequenced [15–18] that provided valuable resources for genome-wide comparative analysis of transposons. Despite 80% of the barley genome consist of various transposons [15], active transposons in barley are still unexplored.

## Results

### Identification of TE-related cDNAs

The availability of barley full-length cDNA (FLcDNA) database [19] provides an invaluable resource for barley genomics. As some transposons are still expressed and can generate mRNAs in host cells [20], we assumed that the barley FLcDNA database may contain TE-related sequences including both expressive transposons and genes containing transposons or fusion transcripts. To test this hypothesis, we used the reported transposase proteins as the queries to search against the 24,783 barley FLcDNA sequences [19], 71 unique FLcDNAs were identified to show significant similarity (E-value < 1 × e − 10) to the transposase proteins of nine extant transposon superfamilies (Table 1, Table S1). The sizes of these FLcDNA sequences varied from 803 bp to 5,883 bp and they were expressed in various types of barley tissues including malting seed, shoot, root, and flower as well as the shoot and root treated with salt, aluminium, abscisic acid (ABA) or jasmonic acid (JA) (Table S1). LTR retrotransposons, non-LTR retrotransposons and DNA transposons contributed 75%, 0.3% and 5% of the barley genome, respectively [15]. However, our results indicated that about 31% (22/71*100) and 44% (31/71*100) of the 71 FLcDNAs were related to LINEs and DNA transposons, and only 25% of them were related to LTR retrotransposons.

**Table 1:** Summary of transposon-related FLcDNAs in barley

Transposon superfamilies	Sequence/transposon name	No. of significant cDNA	Download website
Ty1-Copia LTR retrotransposon	P04146	7	https://www.ncbi.nlm.nih.gov
Ty3-Gypsy LTR retrotransposon	AAC82604	11	https://www.ncbi.nlm.nih.gov
LINEs	AB081316	22	https://www.ncbi.nlm.nih.gov
hAT	AAP54387	10	https://www.ncbi.nlm.nih.gov
Mutator	AAA21566	8	https://www.ncbi.nlm.nih.gov
Helitron	HELITRON7_OS	4	https://www.girinst.org/repbase
CACTA	ENSPM-6_ZM	5	https://www.girinst.org/repbase
Pong	Pong (BK000586)	3	https://www.ncbi.nlm.nih.gov
Tc1/mariner	AAL69341	1	https://www.ncbi.nlm.nih.gov

### FLcDNAs directly derived from intact transposons

**Fig. 1:**
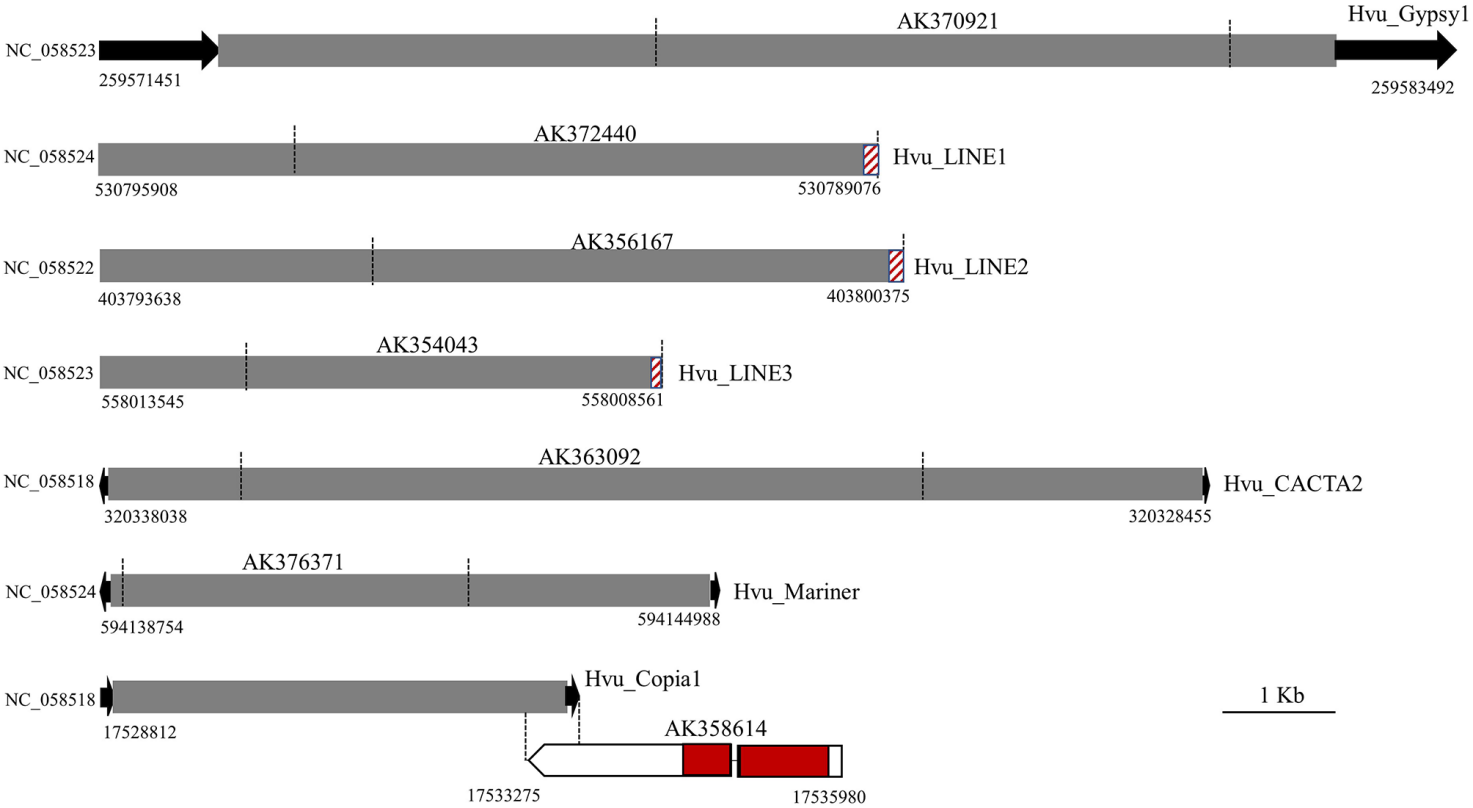
The structures of seven new transposons identified in this study. The black arrows indicate the terminal sequences of transposons including TIRs (opposite orientation) and LTR (same orientation). The brown stripe pattens mean the 3’ poly(A) of LINEs. The black vertical broken lines represent the overlapped regions of transposons and related FLcDNAs. The UTRs and CDSs of gene were indicated by white and red boxes

To investigate if the FLcDNAs were generated by transposons, all 71 FLcDNA sequences listed in Table S1 were used as queries to search against the genome of a malting barley cultivar ‘Morex’ [15], hereafter referred to as the barley reference genome. The significant hits and their 20-Kb flanking sequences (10-kb for each side) were extracted and used to define intact transposons based on target site duplications (TSDs) and the terminal features of different transposon superfamilies including inverted tandem repeats (TIR), LTRs, and 3′ poly(A) tract. No complete transposons were found for 63 FLcDNAs implying they were generated by truncated TEs or the genes harboring some transposon-related sequences. Impressively, we were able to identify eight structurally intact transposons which contain the typical terminal sequences and were flanked by different TSDs. Among these intact transposons, one mutator-like transposon called Hvu_Abermu has been reported in our previous study [21], and other seven, including two LTR retrotransposons, three LINEs and two DNA transposons, were newly identified elements. These transposons ranged in size from 4,949 bp (Hvu_Copia1) to 12,042 bp (Hvu_Gypsy1) and shared 97–100% sequence identity to the related FLcDNAs (Fig. 1, Table S2). Sequence alignments indicated that six intact transposons contained the entire related FLcDNA sequences suggesting they directly generated the full-length transcripts (Fig. 1). However, Hvu_Copia1 overlapped the 3’ untranslated region (UTR) of the FLcDNA, AK358614, that encodes indole-2-monooxygenase-like protein. We further searched against the barley expressed sequence tags (ESTs) deposited in GenBank, eight ESTs showing over 98% sequence identity to the LTRs of Hvu_Copia1 were detected, but no highly identical EST was found for its internal region. Thus, the transcript of AK358614 was likely driven by nearby gene promoter but not from Hvu_Copia1. It seems that Hvu_Copia1 has accumulated mutations or were undergone internal deletions as its predicted proteins lack RNAse H (RNH) and integrase (IN) domains that are necessary for the movement of retrotransposons.

### Transcriptional activity of intact barley transposons

**Fig. 2:**
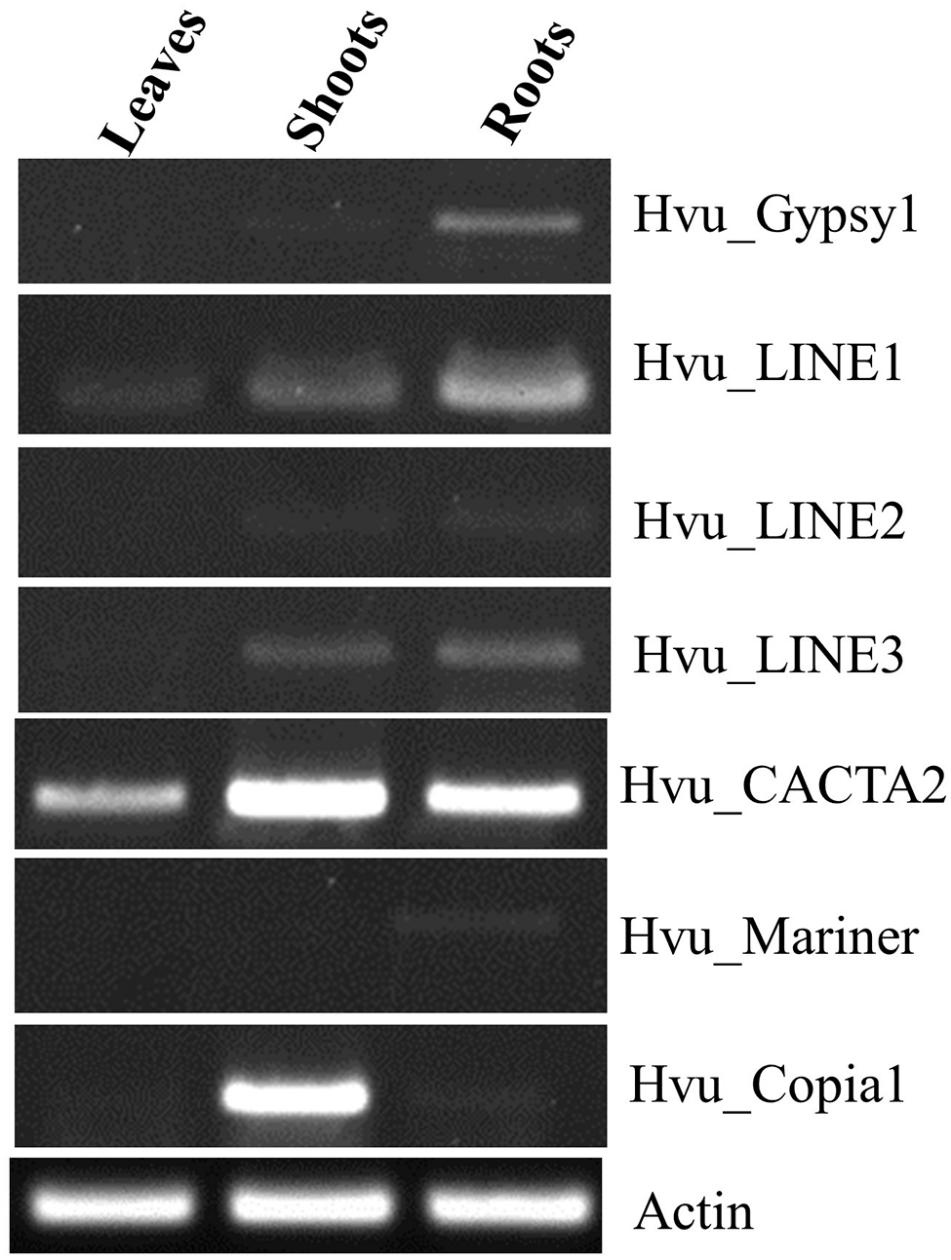
RT-PCR analysis of six transposons in the three types of barley tissues. This figure was cropped from the full-length gels which are shown in Supplementary Material: Figure S1, 2

To validate the transposon expression, we carried out reverse transcription-PCR (RT-PCR) analysis using the primers targeting the overlapped regions between transposons and their related FLcDNAs (Supplementary Table S3). Visible bands with expected amplification sizes were detected for all six transposons (Fig. 2, Fig.S1). Our RT-PCR results also revealed various expression patterns of different transposons. Hvu_CACTA2 and Hvu_LINE1 can generate transcripts in the mRNA samples from leaves, shoots, and roots of 3-week barley seedlings but stronger amplification band was observed for Hvu_CACTA2. For Hvu_Mariner, only a weak band was amplified in the roots and no visible band was found in the young leaves and shoots. For Hvu_LINE2, Hvu_LINE3 and Hvu_Gypsy1, visible bands were observed in the mRNA samples from two shoot and root tissues but not in the leaves (Fig. 2). Compared to the barley FLcDNA database [19], our results confirmed the expression of six intact transposons but showed some new expression patterns. For instance, Hvu_CACTA2 that generated the FLcDNA (AK363092) was expressed in the seedling shoot and root with ABA treatment (Table S1). We detected the expression of Hvu_CACTA2 in leaves, shoots, and roots of seedlings without any stress or treatment. For Hvu_Mariner which generated the FLcDNA (AK376371) and was expressed in the young flower (Table S1), but we also observed its expression in the seedling roots.

### Recent mobility of intact barley transposons

**Fig. 3:**
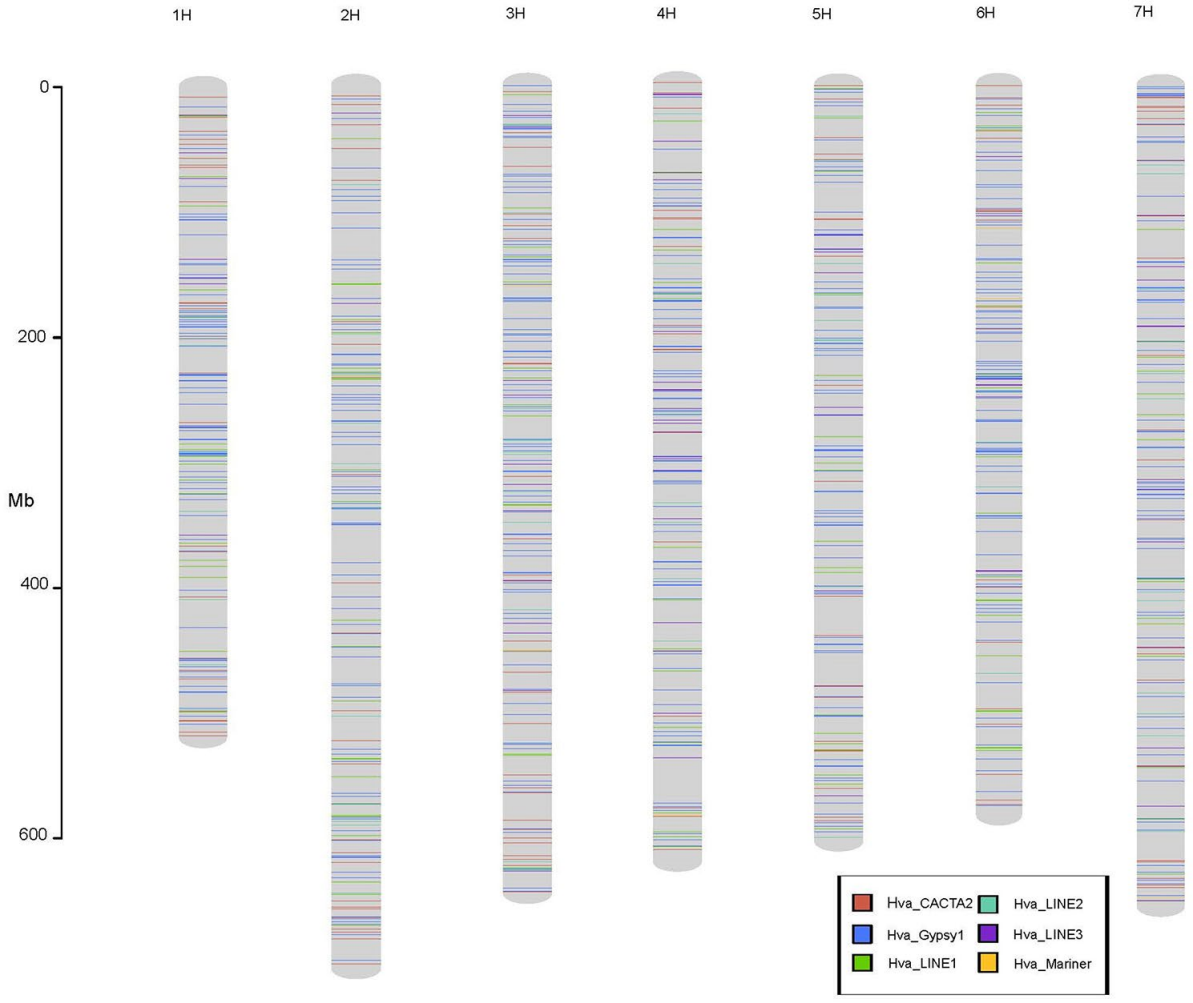
Distributions of six transposon families on seven chromosomes of ‘Morex’

Our sequence analysis and RT-PCR experiment indicated the transcriptional activity of six intact barley transposons. To test if these transposons were recently active, the six transposons, Hvu_Gypsy1, Hvu_LINE1, Hvu_LINE2, Hvu_LINE3, Hvu_CACTA2 and Hvu_Mariner, were combined and used as the library to screen the barley reference genomes [15] with the RepeatMasker software (https://www.repeatmasker.org). Multiple hits were found for all these six transposon families, and the repetitive sequences were dispersed across all seven barley chromosomes but not concentrated in some specific regions (Fig. 3). However, the copy numbers were dramatically different for the six TE families suggesting they had distinct success in the barley genome. 33,833 and 31,562 hits were found for Hvu_Gypsy1 and Hvu_CACTA2, respectively. However, only 628 repeats of Hvu_Mariner were identified. In addition, 2,656, 1,608 and 1,811 repeats were detected for Hvu_LINE1, Hvu_LINE2, and Hvu_LINE3. Most of these repeats were fragmental and/or lacked the typical terminal sequences such as TIRs, LTRs and 3′ poly(A) tract. However, we were able to define the exact boundaries for 190 elements from the six transposon families based on TSDs, terminal motifs and the alignments with the reference transposons.

**Fig. 4:**
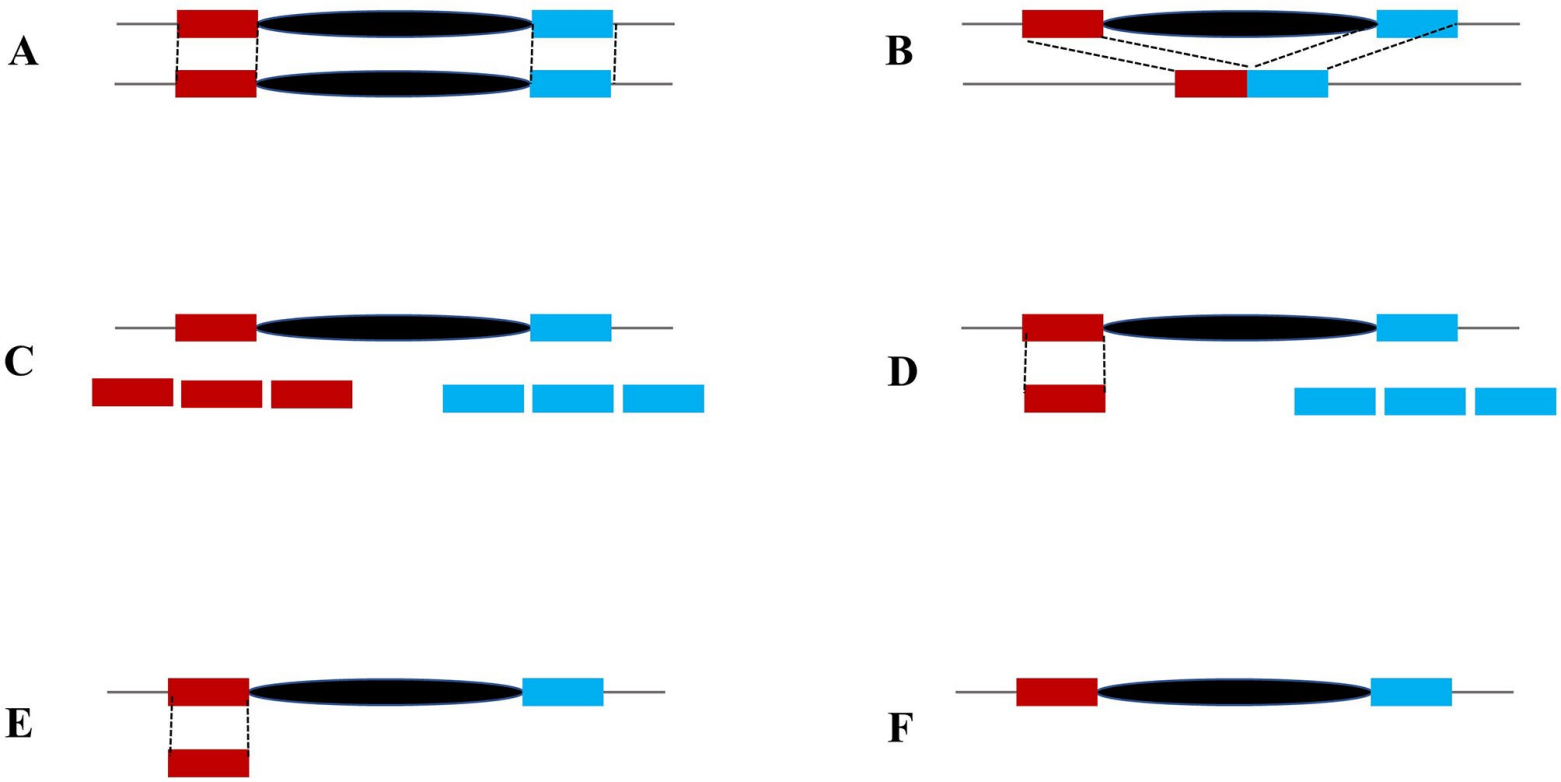
The comparison of transposons between ‘Morex’ and other three barley genomes. The 350-bp right (red box) and left (blue box) flanking sequence of a transposon (black oval) was extracted from the ‘Morex’ genome and merged and used to search against other genomes and define the orthologous sequences. (**A**) Both transposon and the flanking sequences were identified in ‘Morex’ and other genomes. (**B**) Orthologous flanking sequences were identified in another genome, but no transposon was found between the right and left flanking sequences. (**C**) Multiple hits with same sequence identity were found for both flanking sequences in another genome. (**D**) One orthologous hit was defined for one flanking sequence, but multiple hits were identified for another flanking region. (**E**) One orthologous hit was defined for one flanking sequence, but no hit was identified for another flanking sequence. (**F**) no hit was identified for both flanking sequences

The genomes of multiple cultivated and wild barley accessions have been sequenced [15–18], it provided good resources for conducting genome-wide transposon comparison and identifying recently active transposons. To test if the six transposon families were recently active, the 700-bp flanking sequences (350-bp for each side) of the 190 transposons with well-defined boundaries in the reference barley genome [15] were used to align the genomes of two barley varieties, the malting barley ‘Golden Promise’ in the United Kingdom [16, 17] and the Tibetan hulless food barley ‘Lasa Goumang’ in China [18], and the wild barley (*Hordeum spontaneum*, B1K-04-12) [16]. 145 elements were shared between the genomes ‘Morex’ and ‘Golden Promise’ as the transposons and flanking sequences were found in both genomes (Fig. 4A). Four polymorphic transposons, including one Hvu_LINE1, one Hvu_LINE3 and two Hvu_Mariner, were detected as the specific flanking sequences of these four elements identified in the ‘Golden Promise’ genome, but no transposon was found between the right and left flanking sequences (Fig. 4B). However, we were not able to determine the presence or absence of other 41 elements in the genome of ‘Golden Promise’ as no hits or multiple hits with same sequence identity were detected in ‘Golden Promise’ (Fig. 4C-F) implying that these elements were inserted into repetitive genomic regions, or the flanking were not assembled into the genome. The flanking sequences of 190 elements in ‘Morex’ also were used to conduct BLASTN searches against the genome of the ‘Lasa Goumang’, 136 elements were shared between ‘Morex’ and ‘Lasa Goumang’, nine elements, including three Hvu_LINE3 retrotransposons and six Hvu_Mariner transposons, were present in the genome of ‘Morex’ but absent in the genome of ‘Lasa Goumang’. The presence/absence of 46 elements were not clear in the genome of ‘Lasa Goumang’ as no specific hits were identified for their flanking sequences. By comparing the genomes of ‘Morex’ and ‘B1K-04-12’, 131 shared transposons were identified, and 10 elements, including one Hvu_LINE1, three Hvu_LINE3 and six Hvu_Mariners, were absent in the wild barley genome. The detailed transposon comparisons between the reference barley genome and other three genomes are shown in the Table S4. Overall, our genome-wide transposon comparisons revealed the polymorphisms of three transposon families, Hvu_LINE1, Hvu_LINE3 and Hvu_Mariner, between ‘Morex’ and other three barley genomes suggesting their recent transposition or retrotransposition activity occurring after the barley domestication.

### Contributions of transposons on barley genes

**Fig. 5:**
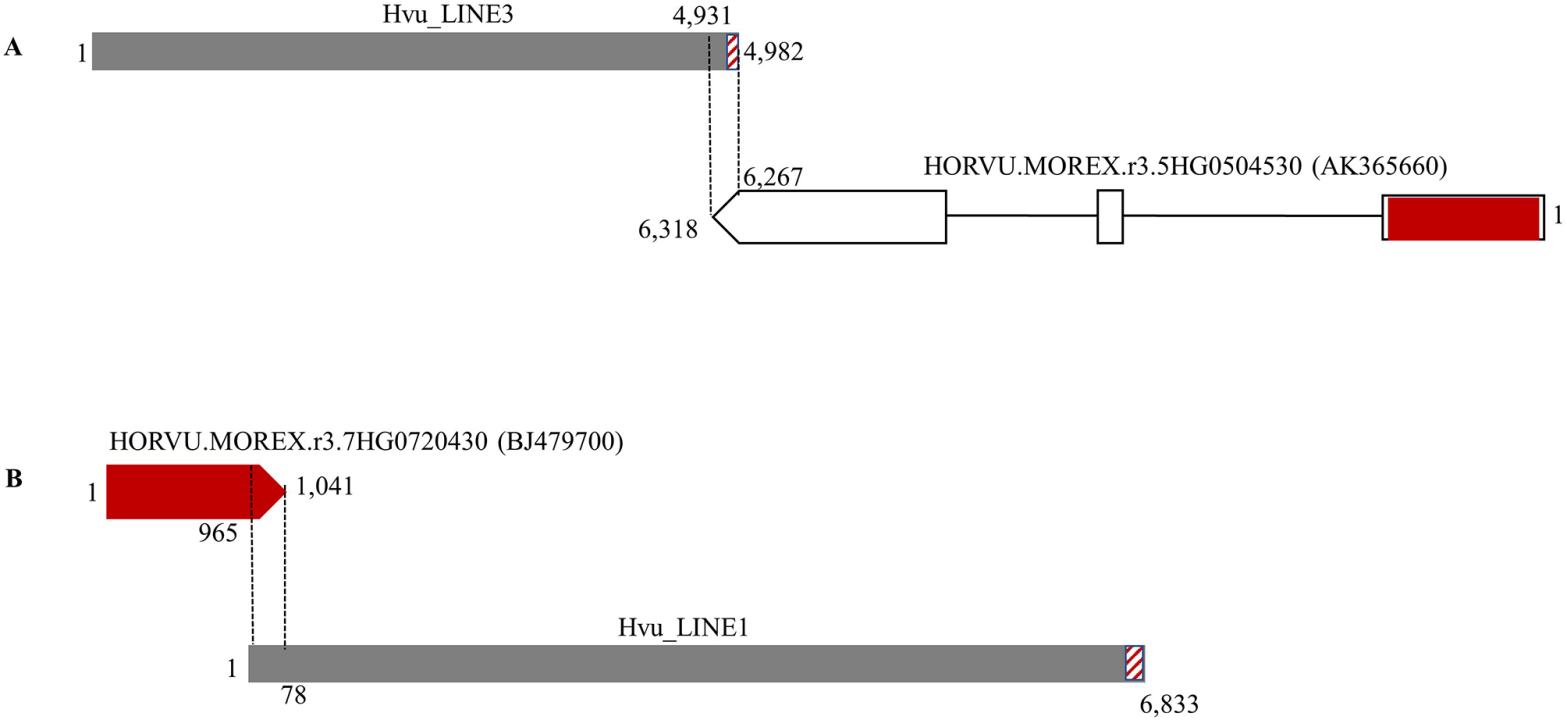
Two annotated barley genes for which transposon was recruited as the 3’UTR (**A**) and CDS (**B**), respectively. The red and white boxes represent the CDSs and UTRs of genes. The black vertical lines mean the overlapped regions between the genes and transposons. The GenBank accession number of FLcDNA or EST that supports the gene model and expression is shown in () after the gene name

Transposons are important sources of gene evolution as their movements can result in gene mutation, alter gene expression, or serve as genic sequences or regulatory elements [22, 23]. To detect if the six identified transposons were recruited as exons of barley genes, we searched against the transcripts of all annotated barley genes [15] using the six transposons as the queries. A total of 213 annotated genes showed significant identity to the six transposons indicating their transcripts contained transposon-related sequences. To gain insights into their functional information, the transcripts of all 213 genes were used to search against the GenBank non-redundant protein sequence database. 81 genes encoded proteins containing the conserved transposase or retrotransposase domains implying that they were likely involved in catalysing transposon movement. 85 genes were annotated as hypothetical protein or uncharacterized protein, or their functions were not defined. Interestingly, 47 genes with various molecular functions, including disease resistance and development regulation, were identified (Table S5). Further sequence comparisons indicated that the UTRs of nine genes contained transposon sequences including *HORVU.MOREX.r3.5HG0504530* encoding protein ELC-like (Table S5; Fig. 5A). However, for most of the genes (38/47 = 80.9%), transposons were recruited as the coding sequences (CDSs) of barley genes including the gene *HORVU.MOREX.r3.7HG0720430* encoding F-box/LRR-repeat protein 13-like (Table S5; Fig. 5B).

## Discussion

Maintenance of transposon activities is extremely important for their long-term survivals in host genomes [24]. However, active transposons are unusual in all sequenced genomes as their mobility are strictly suppressed by the epigenetic machinery [6] and they can also be eliminated from host genomes over time by the purifying selection [25]. Therefore, active TEs provide valuable resources for investigating the transposition mechanisms of different transposon families and addressing the molecular system by which transposons can exploit to evade the epigenetic regulations. Additionally, active transposons are invaluable for understanding gene functions and developing molecular tools [26]. The barley genome is large (~ 5.1 Gb) and comprises about 80% of transposons [15]. However, characterization of active transposons in barley is still overlooked. Thus far, all transposon-based genetic resources for barley research were developed with the maize Ac/Ds transposons [27]. In this study, we conducted genome-wide comparative analysis and detected polymorphisms for three TE families within three cultivated barley varieties and between cultivated and wild barley genomes suggesting these transposons were active after the domestications of cultivated barley or they may be still active in the cultivated barley populations. Despite more molecular experiments are needed to confirm their mobility by identifying polymorphic insertions between parents and the offspring or between the wild types and regenerated plants derived from tissue culture and/or other stresses, the three transposon families, Hvu_LINE1, Hvu_LINE3 and Hvu_Mariner, and Hvu_Abermu which was identified in our previous study [21] offer helpful information and the potential sources to develop new molecular markers and genetic resources such as endogenous gene tagging system for barley functional gene studies.

Thus far, many computational software has been developed for genome-wide transposon annotation. However, it still is challenging for characterizing active transposons. Some active transposons were identified with molecular methods as they inserted into functional genes and caused phenotypical variations [13, 28]. However, visible mutations related to transposons were infrequently as many transposons inserted into intergenic regions and cannot change the morphological traits. Additionally, it is time-consuming to clone functional genes. The public availability of genome sequences allows us to conduct genome-wide transposon comparisons and identify the new and recent TE insertions. The efficiency of this approach heavily depends on high quality genomes and transposon annotation, especially well-defined transposon boundaries, as well as well sequenced transcriptomic databases. As some transposons can be reactivated by tissue culture or other stressful treatments [29] and generated transcripts, active transposons can also be discovered by analyzing transcript profiling of transposons of plant calli [30]. In this study, we analyzed the barley FLcDNA database and identified seven intact transposons showing significant sequence identity to the reported FLcDNA sequences. Our RT-PCR experiment also confirmed their expression (Fig. 2) suggesting the transcriptional activity of these transposons. The genome-wide comparisons of six transposon families detected polymorphisms of three TE families. Thus, our method by comparing both transcriptional and genomic data sets can be used to identify recently active TEs in other genomes. Anderson et al. analyzed 359 RNAseq libraries of B73 inbred line collected from diverse set of tissues and developmental stages and found that TEs contributed 1.4–26.1% of the reads assigned to genes or TEs [31]. In migratory locust (*Locusta migratoria*), transposon-related transcripts can comprise about 20% of the transcriptome [32]. However, we only found 0.3% (70/24,783*100) of barley FLcDNAs were related to transposons, the rate of transposon-related transcripts was much lower than that in maize and migratory locust. It may suggest different transcriptional landscapes between different organisms. Another possible reason was that many transposon-related transcripts were ruled out by the FLcDNA database [19]. As the expression of genes and transposons heavily dependents on the tissue types, growth stages, and growth/treatment conditions, many transposon-related transcripts may not be generated yet. Additionally, many intact transposons are suppressed by the host’s the epigenetic machinery [6], and they can only be expressed under tissue culture or other stress treatments or in the plants with methylation mutations such as ddm1 mutant in Arabidopsis in which many silenced can be reactivated and expressed [33]. In our next work, we will analyze barley long-read transcriptome dataset from more diverse tissues, developmental stages and growth conditions, and gain more clues about the transposon transcripts in barley. Though our method may not be suitable to find active nonautonomous TEs, but it provides an approach to identify chimeric transcripts containing both genes and TEs by screening the barley transcriptome dataset. It should be noted that some transposon-related transcripts may still can be missed from large transcriptomic databases, but they can be detected by RT-PCR analysis. Thus, it is necessary to combine computational and experimental methods to efficiently identify active transposons and transposon-related transcripts.

Due to the potential harmful impacts [4, 5], transposons mostly concentrated in heterochromatic regions or intergenic regions and the deleterious insertions can be rapidly eliminated from host genomes [25]. However, transposons also play critical roles in gene and genome evolution as they provide raw materials for generating new genes or novel phenotypic mutations [34] and can affect the transcription of nearby host genes. For example, the insertion of a LTR retrotransposon in the promoter of VvmybA1 gene induced the variations of grape skin colors [35]. The transposons located in introns may change splice sites and affects the epigenetic landscape of the gene [36] and many transposons, especially the smaller TEs such as miniature inverted-repeat transposable elements (MITEs) and terminal-repeat retrotransposons in miniature (TRIMs), were frequently found in intronic regions [37]. However, it was very rare to identify transposons in exons. Impressively, we identified 213 genes which transcripts showed significant sequence identity to the six transposons. Most of these genes likely encode transposase proteins or other products with undefined functions. However, 47 genes with different functions were found. Nine genes contain TEs serving as UTRs and 38 genes harbor transposons serving as CDSs. Previous studies suggested that some transposons can be ‘domesticated’ or undergo co-option and provide new cellular functions including the yield traits in rice (*Oryza sativa*) [38]. It is needed to further characterize the molecular functions of these 47 barley genes and validating the gene annotations.

Despite some transposons can maintain their activity for multiple generations [39]. In most cases, active transposons cannot be maintained for long period due to the epigenetic regulations in host cells. We analyzed the barley FLcDNA database which was generated from a Japanese malting barley variety ‘Haruna Nijo’ [19] and identified related FLcDNAs for seven transposons suggesting these transposons may still be transcriptional active in ‘Haruna Nijo’. We were not able to collect the seeds of ‘Haruna Nijo’, but we confirmed their expression with an American barley variety ‘Morex’. Thus, our computational and molecular analysis indicated that the six transposons are likely expressed in multiple barley varieties under natural condition. We also searched against the wheat (*Triticum aestivum*) expression database and identified highly significant hits (E-value < 1 × e − 90) for all the seven TEs but Hvu_LINE1 and Hvu_Mariner suggesting they are likely transcriptional active in wheat. How these transposons maintain their activity in barley? Which mechanism they are using to evade the host regulation? All these questions are necessary to be addressed in our later studies.

## Conclusions

In this study, we conducted computational and molecular analysis and identified six new transcriptional TEs in barley. The RT-PCR results indicated differential expression of the transposons. The genome-wide transposon comparisons revealed the recent mobility of three transposons after the barley domestication. We also detected TE-related sequences serving as UTRs or CDSs of the annotated barley genes. Overall, our efforts provide important resource for addressing the transposon activities and for developing genetic tools for barley improvement and other related studies.

## Materials and methods

### Materials

The seeds of a malting barley variety ‘Morex’ (Accession number: CIho 15,773) were obtained from the National Small Grains Collection at USDA-ARS Small Grains and Potato Germplasm Research Unit. The seeds were germinated and grown at room temperature in vermiculite for 3 weeks.

### Computational analysis

#### Data sets

All barley full-length cDNAs (FLcDNAs) sequences were published by Matsumoto et al. [19]. The barley refence genome [15] was obtained from the Ensembl Project website (http://plants.ensembl.org/info/about/index.html). The genomic sequences of Tibetan naked barley ‘Lasa Goumang’ [18] were downloaded from NCBI (GenBank accession numbers: SDOW01000001-SDOW01001856). Additionally, the genomes of ‘Golden Promise’ (*Hordeum vulgare*) and B1K-04-12 (*Hordeum spontaneum*) were downloaded from the barley pan-genome website (10.1038/s41586-020-2947-8) [16].

### Transposon identification

To detect expressed transposon-related sequences, we conducted TBLASTN search against the barley full-length cDNAs (FLcDNAs) database with the transposase proteins of nine extant transposon superfamilies (Table 1). All significant hits (E-value < 1 × e − 10) were manually inspected to remove the redundancy sequences. The unique FLcDNAs were used as the queries to conduct BLASTN searches against the barley reference genome [15]. The genomic sequences showing significant identity (E-value < 1 × e − 10) to the FLcDNAs for over 50-bp matched regions were kept. We next extracted the hits and their 20-Kb flanking sequences (10-Kb for each side) and performed all against all sequence alignments for determining the transposon boundaries based on the terminal repeats, target site duplications.

### Genome-wide transposon comparisons

The intact transposons were used as the library to screen the barley reference genome [15] with RepeatMasker program (https://www.repeatmasker.org) using the default parameters but we applied the “nolow” option to avoid masking the low-complexity DNA. All hits were manually curated to find the elements which boundaries can be clearly determined based on the terminal sequences, flanking TSDs and sequence comparisons with the reference element. The 700-bp flanking sequence (350-bp for each side) for each transposon with well-defined boundaries was used to search against the genomes of ‘Golden Promise’ [16, 17], ‘Lasa Goumang’ [18] and the wild barley B1K-04-12 [16] and to detect its presence/absence in three barley genomes based on the sequence alignments.

### Visualization of transposon distributions

To visualize the distributions of different transposon families on seven barley chromosomes, the RepeatMasker output file was converted into an Excel file and used to display the genomic data with chromPlot software [40].

### Reverse transcription PCR (RT PCR)

The leaves, stems and roots of the Morex seedlings were collected and quickly frozen in liquid nitrogen. The collected samples were then stored in the − 80 °C freezer. The total RNA was isolated using the Thermo scientific GeneJET RNA purification kit (Thermo Fisher Scientific, Waltham, MA) by following the recommended protocol. The quality of extracted RNA was checked with the 1% agarose gel. Fourteen mg of total RNA from each sample was used to convert RNA into single-strand cDNA using the iScript gDNA Clear cDNA Synthesis kit (Bio-Rad Laboratories, Hercules, CA) by following the protocol. One µl of the cDNA was used to run PCR with the barley actin gene primers [actinF (5′- CCCAAAAGCCAACAGAGAGA-3′) and actinR (5′- GCCTGAATAGCGACGTACAT-3′)] [41] and 10 µl of the PCR products were used for quantitative comparison of mRNA levels, only the samples with similar concentrations were used for RT-PCR analysis of transposons.

RT-PCR primers (Supplementary Table S3) were designed based on the transposon-related full cDNA sequences (Fig. 1) with the Primer3 software [42]. The designed primers were synthesized by the Eurofins Genomics LLC (Louisville, KY, United States). PCR amplifications were conducted in a Bio-Rad S1000 Thermal Cycler in 20 µl reactions consisting of 1 µl of cDNA, 0.2 mM primer, deionized water, and 10 µl EconoTaq PLUS GREEN 2X Master Mix (Middleton, WI) containing 0.1 units/µl of EconoTaq DNA Polymerase, Reaction Buffer (pH 9.0), 400 µM dATP, 400 µM dGTP, 400 µM dCTP, 400 µM dATP, and 3 mM MgCl2. The PCR temperature cycling conditions were 1 cycle of 98 °C for 2 min; 30 cycles of 95 °C for 30 s, 55 °C for 30 s, 72 °C for 30 s; and 1 cycle of 72 °C for 5 min. Amplification products were run on 0.8% agarose gels and stained with ethidium bromide.

### Electronic supplementary material

Below is the link to the electronic supplementary material.

**Supplementary Information: Table S1**. The list of FLcDNAs related to transposons. **Table S2**. Seven intact barley transposons identified in this study. **Table S3**. Summary of primers used for RT-PCR analysis. **Table S4**. Genome-wide transposon comparisons between the ‘Morex’ genome and other three barley genomes. **Table S5**. The list of annotated barley genes which transcripts showed significant sequence identity to the six identified transposons. **Figure S1**. Full-length gel for RT-PCR analysis of barley transposons. **Figure S2**. Full-length gel for RT-PCR analysis of barley actin gene.

## Data Availability

The datasets generated and/or analyzed during the current study are available in the supplementary materials.

## Abbreviations

CDS: Coding region
FLcDNA: Full-length cDNA
EST: Expressed sequence tag
LINEs: Long interspersed nuclear elements
LTR: Long terminal repeat
RT-PCR: Reverse transcription-PCR
SINEs: Short interspersed nuclear elements
TEs: Transposable elements
TIR: Inverted tandem repeat
TSD: Target site duplications
UTR: Untranslated region
