# Supplementary material for: Identification of transcriptionally active transposons in Barley
Source: BMC Genom Data. 2023 Nov 4;24:64. doi: 10.1186/s12863-023-01170-1 (PMC10625261; doi:10.1186/s12863-023-01170-1)
Supplement: Supplementary file 1 — Supplementary Information: Table S1. The list of FLcDNAs related to transposons. Table S2. Seven intact barley transposons identified in this study. Table S3. Summary of primers used for RT-PCR analysis. Table S4. Genome-wide transposon comparisons between the ‘Morex’ genome and other three barley genomes. Table S5. The list of annotated barley genes which transcripts showed significant sequence identity to the six identified transposons. Figure S1. Full-length gel for RT-PCR analysis of barley transposons. Figure S2. Full-length gel for RT-PCR analysis of barley actin gene. [file 12863_2023_1170_MOESM1_ESM.pdf]

Supplementary Information

Table S1. The list of FLcDNAs related to transposons.

Table S2. Seven intact barley transposons identified in this study.

Table S3. Summary of primers used for RT-PCR analysis.

Table S4. Genome-wide transposon comparisons between the 'Morex' genome and other three barley genomes.

Table S5. The list of annotated barley genes which transcripts showed significant sequence identity to the six identified transposons.

Figure S1. Full-length gel for RT-PCR analysis of barley transposons.

Figure S2. Full-length gel for RT-PCR analysis of barley actin gene.

Table S1. The list of FLcDNAs related to transposons

FLcDNA	Size of FLcDNA (bp)	Tissue type for mRNA extraction	Transposons	E-Value
AK372440	5757	malting seed	Karma-AB081316	2E-141
AK356167	5441	seedling shoot in normal or dark	Karma-AB081316	1E-134
AK354043	4771	unknown	Karma-AB081316	4E-129
AK365543	3217	seedling shoot and root in salt treatment or abscission	Karma-AB081316	8E-92
AK376426	4460	unknown	Karma-AB081316	3E-53
AK365531	2699	unknown	Karma-AB081316	7E-50
AK376284	3750	malting seed	Karma-AB081316	7E-44
AK354591	3320	seedling shoot in normal or dark	Karma-AB081316	3E-37
AK363514	4238	seedling shoot and root on Aluminium stress	Karma-AB081316	2E-33
AK363820	2483	seedling shoot and root on Aluminium stress	Karma-AB081316	3E-22
AK357680	5883	unknown	Karma-AB081316	2E-20
AK372062	4347	seedling shoot and root with JA treatment	Karma-AB081316	1E-19
AK366636	3962	seedling shoot and root with ABA treatment	Karma-AB081316	2E-16
AK374558	4099	malting seed	Karma-AB081316	1E-14
AK249188	2006	mixture of seed, leaf, spike, stem and root	Karma-AB081316	3E-14
AK249315	2864	mixture of seed, leaf, spike, stem and root	Karma-AB081316	5E-14
AK356070	2233	seedling shoot in normal or dark	Karma-AB081316	3E-13
AK248406	4451	mixture of seed, leaf, spike, stem and root	Karma-AB081316	1E-11
AK356634	3397	seedling shoot in normal or dark	Karma-AB081316	4E-11
AK364073	2819	seedling shoot and root with JA teatment	Karma-AB081316	4E-11
AK377065	3964	malting seed	Karma-AB081316	6E-11
AK363651	1459	seedling shoot and root in salt treatment or abscission	Karma-AB081316	9E-11
AK370534	2597	seedling shoot and root with JA teatment	hAT	7E-97
AK357459	2720	flag leaf at vegetative stage	hAT	1E-89
AK251912	2614	mixture of seed, leaf, spike, stem and root	hAT	1E-85
AK372382	5232	young panicle	hAT	1E-78
AK370171	3013	seedling shoot and root with JA teatment	hAT	2E-41
AK355471	1364	flag leaf at vegetative stage	hAT	3E-20
AK374079	1243	malting seed	hAT	2E-18
AK366057	2840	seedling shoot and root with ABA treatment	hAT	9E-18
AK365622	2866	seedling shoot and root with JA teatment	hAT	7E-14
AK370562	1967	seedling shoot and root with JA teatment	hAT	4E-13
AK251670	4605	mixture of seed, leaf, spike, stem and root	MudrA	6E-82
AK366163	2661	seedling shoot and root on Aluminium stress	MudrA	5E-61
AK360693	2780	seedling shoot in normal or dark	MudrA	7E-61
AK365336	2760	unknown	MudrA	1E-59
AK374389	2927	adult flower	MudrA	2E-59
AK368677	3155	seedling shoot and root with JA treatment	MudrA	7E-50
AK376919	1887	malting seed	MudrA	5E-27
AK367806	2522	unknown	MudrA	6E-40
AK370391	1846	seedling shoot and root on Aluminium stress	Copia	3E-72
AK362345	1712	seedling shoot and root with ABA treatment	Copia	2E-45
AK375598	3057	adult flower	Copia	1E-51
AK375954	1764	young panicle	Copia	1E-26
AK353758	1441	flag leaf at vegetative stage	Copia	1E-30
AK358614	2555	germinated shoot	Copia	1E-12
AK376516	801	young panicle	Copia	3E-17
AK373117	4525	malting seed	Gypsy	3E-64
AK370921	5158	seedling shoot and root on Aluminium stress	Gypsy	1E-61

AK375533	3788	early flower	Gypsy	5E-55
AK374145	2316	adult flower	Gypsy	4E-51
AK367250	2596	seedling shoot and root in salt treatment or abscission	Gypsy	3E-45
AK368961	2925	seedling shoot and root on Aluminium stress	Gypsy	4E-40
AK376541	3409	malting seed	Gypsy	7E-34
AK250850	3351	mixture of seed, leaf, spike, stem and root	Gypsy	7E-28
AK366907	2957	seedling shoot and root in salt treatment or abscission	Gypsy	2E-23
AK357593	1252	germinated shoot	Gypsy	7E-19
AK371019	2548	seedling shoot and root on Aluminium stress	Gypsy	1E-11
AK376736	3972	malting seed	Pong	2E-49
AK376742	3762	young panicle	Pong	1E-46
AK364564	3188	seedling shoot and root in salt treatment or abscission	Pong	6E-24
AK360890	2229	flag leaf at vegetative stage	Helitron-Os	5E-16
AK373927	1853	malting seed	Helitron-Os	2E-15
AK376804	1826	malting seed	Helitron-Os	5E-15
AK368190	2576	seedling shoot and root with JA treatment	Helitron-Os	3E-14
AK372292	5154	seedling shoot and root with ABA treatment	ENSPM-6_ZM	6E-50
AK363092	5813	seedling shoot and root with ABA treatment	ENSPM-6_ZM	3E-44
AK373539	912	early flower	ENSPM-6_ZM	5E-19
AK373166	1062	young panicle	ENSPM-6_ZM	6E-18
AK248767	1823	mixture of seed, leaf, spike, stem and root	ENSPM-6_ZM	5E-11
AK376371	2237	early flower	mariner-CC	3E-31

Table S2. Seven intact barley transposons identified in this study

Name	Location	Target site duplication	FLcDNAs (Sequence identity to transposon)
Hvu_Copia1	NC_058518 (17528812-17533760)	ACCTG	AK358614 (99%)
Hvu_Gypsy1	NC_058523 (259571451- 259583492)	CCCCC/CCCAC	AK370921 (99%)
Hvu_LINE1	NC_058524 (530795908-530789076)	CACATTGGTACCAA	AK372440 (99%)
Hvu_LINE2	NC_058522 (403793638-403800375)	CAACCGAAGTTCC	AK356167 (99%)
Hvu_LINE3	NC_058523 (558013545-558008561)	CTGTTGTGTTTT	AK354043 (99%)
Hvu_CACTA2	NC_058518 (320338038-320328455)	ATG/AGG	AK363092 (97%)
Hvu_Mariner#	NC_058524 (594138754-594144988)	TA	AK376371 (100%)

Table S3. Summary of primers used for RT-PCR analysis

Transposon	Forward primer (5' to 3')	Reverse primer (5' to 3')
Hvu_Gypsy1	CAATACAGCCCTTCAAACCAA	GATTGACGCGCTCTCTATGTC
Hvu_LINE1	CGATCATGTGTTGGAACGTC	GTGGGAGGTTACGGTAGCAA
Hvu_LINE2	GCGTGCATAGCCTCCTAGTC	AACCTTCGCGAATAACATGG
Hvu_LINE3	CCGACGATGTGATGTTGTTC	CTGCTTCAGGGTTTTCTTCG
Hvu_CACTA2	TATTCGCCAATAATGTTTGTT	TGGGTTTTTACTTCATCGTAA
Hvu_Mariner	CTGGAAAGTGACCCTAGAAAG	CATCTTCTCTCCTCTCTCCTC

Table S4. Genome-wide transposon comparisons between the 'Morex' genome and other three barley genomes

Complete transposons in the Morex genome				Distributions in Goden Promise	Distributions in Lasa Goumang	Distributions in B1K-04-12
Chromosome	Start	End	Transposon	P: presence; a: absence	P: presence; a: absence	P: presence; a: absence
1H	309844407	309863316	Hvu_LINE2	p	p	p
3H	602481585	602488381	Hvu_LINE2	p	p	p
4H	17197483	17204196	Hvu_LINE2	p	p	p
4H	260023693	260030412	Hvu_LINE2	p	p	p
4H	437686813	437693681	Hvu_LINE2	p	p	p
5H	59377972	59384695	Hvu_LINE2	p	p	p
5H	403793638	403800375	Hvu_LINE2	p	p	p
1H	171143317	171149935	Hvu_LINE3	?	p	p
1H	355945460	355951944	Hvu_LINE3	p	p	p
1H	510942582	510949160	Hvu_LINE3	p	p	p
2H	75899288	75905812	Hvu_LINE3	p	p	p
2H	114606392	114612986	Hvu_LINE3	p	p	p
2H	163879858	163886490	Hvu_LINE3	p	p	p
2H	552394890	552401409	Hvu_LINE3	p	p	p
3H	239110369	239117047	Hvu_LINE3	p	p	p
3H	446476587	446482312	Hvu_LINE3	p	p	p
3H	452403435	452409963	Hvu_LINE3	p	p	p
4H	9990995	9997587	Hvu_LINE3	p	p	a
4H	97443163	97449363	Hvu_LINE3	p	p	p
4H	321266949	321272923	Hvu_LINE3	p	p	p
4H	525572333	525578912	Hvu_LINE3	a	p	a
4H	536004470	536010955	Hvu_LINE3	p	p	p
5H	38528988	38535643	Hvu_LINE3	p	a	p
5H	130102297	130107394	Hvu_LINE3	p	p	p
5H	230334260	230339857	Hvu_LINE3	p	p	p
5H	354880020	354886302	Hvu_LINE3	p	p	p
6H	17790135	17795216	Hvu_LINE3	p	?	p
6H	40106704	40113137	Hvu_LINE3	p	p	p
6H	101811684	101816090	Hvu_LINE3	p	p	p
6H	101821640	101823412	Hvu_LINE3	p	p	p
6H	102715033	102721706	Hvu_LINE3	p	p	p
6H	170799270	170805905	Hvu_LINE3	p	p	p
6H	262353640	262360246	Hvu_LINE3	p	p	p
6H	281126061	281132381	Hvu_LINE3	p	p	p
6H	492312397	492318808	Hvu_LINE3	p	p	p
6H	558008561	558013545	Hvu_LINE3	p	a	p
7H	524754007	524760627	Hvu_LINE3	p	a	a
7H	590503300	590509914	Hvu_LINE3	p	p	p
5H	375591268	375598029	Hvu_LINE1	?	p	a
6H	316513894	316520702	Hvu_LINE1	p	p	p
7H	439825347	439832141	Hvu_LINE1	p	p	p
7H	530789076	530795908	Hvu_LINE1	a	p	p
6H	259571451	259583705	Hvu_Gypsy1	p	p	p
1H	320328455	320338038	Hvu_CACTA2	?	?	?
3H	579021343	579044428	Hvu_CACTA2	?	?	?
2H	52277554	52323535	Hvu_CACTA2	?	?	?
1H	9896016	9896306	Hvu_Mariner	p	p	p
1H	24028919	24029208	Hvu_Mariner	p	p	p
1H	167266599	167266888	Hvu_Mariner	?	?	?
1H	231890183	231890463	Hvu_Mariner	p	p	p
1H	253898977	253899266	Hvu_Mariner	?	?	?
1H	256966147	256966437	Hvu_Mariner	p	p	p
1H	283262758	283263048	Hvu_Mariner	p	p	p
1H	407186484	407187105	Hvu_Mariner	p	p	p
1H	409604413	409604704	Hvu_Mariner	p	p	p
1H	456802705	456802994	Hvu_Mariner	p	p	p
1H	476068598	476068886	Hvu_Mariner	p	p	p
1H	488344399	488344689	Hvu_Mariner	p	p	?
1H	512626967	512627253	Hvu_Mariner	p	p	?
2H	2280620	2280911	Hvu_Mariner	p	?	p
2H	4829879	4830170	Hvu_Mariner	?	?	?
2H	19087776	19088067	Hvu_Mariner	p	?	p
2H	34802410	34802701	Hvu_Mariner	p	a	a
2H	55214428	55214719	Hvu_Mariner	p	?	p
2H	63943194	63943485	Hvu_Mariner	p	p	p
2H	130860135	130860424	Hvu_Mariner	p	p	p
2H	132556833	132557124	Hvu_Mariner	?	?	?
2H	172955654	172957938	Hvu_Mariner	p	p	p
2H	180627219	180629506	Hvu_Mariner	p	p	p
2H	222083611	222087337	Hvu_Mariner	?	?	?
2H	277018467	277021438	Hvu_Mariner	p	p	p
2H	282131346	282131633	Hvu_Mariner	p	p	p
2H	395671441	395671733	Hvu_Mariner	p	p	p
2H	432271982	432276585	Hvu_Mariner	p	p	?

2H	450789223	450790377	Hvu_Mariner	a	?	a
2H	507577985	507578275	Hvu_Mariner	p	p	p
2H	508630018	508630309	Hvu_Mariner	?	?	?
2H	511810085	511810376	Hvu_Mariner	p	p	p
2H	563380506	563380797	Hvu_Mariner	p	p	p
2H	565964831	565969195	Hvu_Mariner	?	?	a
2H	567833432	567833723	Hvu_Mariner	p	p	p
2H	583099723	583100014	Hvu_Mariner	p	p	p
2H	584874148	584874439	Hvu_Mariner	p	p	p
2H	599546736	599547027	Hvu_Mariner	p	p	p
2H	600592564	600592959	Hvu_Mariner	?	?	?
2H	611461194	611461485	Hvu_Mariner	p	p	?
2H	612870461	612870752	Hvu_Mariner	p	p	p
2H	613485486	613485776	Hvu_Mariner	p	p	p
2H	619622251	619622542	Hvu_Mariner	p	p	p
2H	636848116	636848404	Hvu_Mariner	p	p	p
2H	638565220	638565510	Hvu_Mariner	p	p	p
2H	643299904	643300193	Hvu_Mariner	p	?	p
2H	646283527	646283818	Hvu_Mariner	p	p	p
3H	5424610	5424977	Hvu_Mariner	p	?	p
3H	14024319	14027144	Hvu_Mariner	p	p	p
3H	23437953	23438242	Hvu_Mariner	?	?	?
3H	137490381	137490673	Hvu_Mariner	p	p	p
3H	155353654	155355042	Hvu_Mariner	p	p	p
3H	445980551	445980842	Hvu_Mariner	p	p	p
3H	487857072	487858350	Hvu_Mariner	p	p	p
3H	502632178	502632469	Hvu_Mariner	?	p	?
3H	511562408	511562699	Hvu_Mariner	p	p	?
3H	523074104	523074502	Hvu_Mariner	p	?	?
3H	529455651	529455941	Hvu_Mariner	?	p	?
3H	548171429	548171720	Hvu_Mariner	p	p	p
3H	555322919	555323316	Hvu_Mariner	a	a	a
3H	561061219	561061508	Hvu_Mariner	p	p	p
3H	561394327	561394696	Hvu_Mariner	?	p	?
3H	565616716	565617005	Hvu_Mariner	p	p	p
3H	595292113	595292406	Hvu_Mariner	?	?	?
4H	24599428	24599718	Hvu_Mariner	p	?	?
4H	26826416	26826706	Hvu_Mariner	p	p	p
4H	41761789	41762078	Hvu_Mariner	?	?	?
4H	97522372	97522772	Hvu_Mariner	?	?	?
4H	100213458	100213748	Hvu_Mariner	p	p	p
4H	161207113	161207422	Hvu_Mariner	p	p	p
4H	219222437	219222726	Hvu_Mariner	p	p	p
4H	253893232	253893522	Hvu_Mariner	?	?	?
4H	321755818	321756107	Hvu_Mariner	p	p	p
4H	381751603	381751894	Hvu_Mariner	?	?	?
4H	430743229	430747062	Hvu_Mariner	p	p	p
4H	483647398	483647689	Hvu_Mariner	p	p	p
4H	495244761	495245052	Hvu_Mariner	p	p	p
4H	504707047	504711662	Hvu_Mariner	p	p	p
4H	522389719	522390009	Hvu_Mariner	p	p	p
4H	542724302	542724591	Hvu_Mariner	?	?	?
4H	549537977	549538266	Hvu_Mariner	?	p	?
4H	560927586	560927877	Hvu_Mariner	p	p	p
4H	574403961	574407327	Hvu_Mariner	p	p	p
4H	581175448	581175528	Hvu_Mariner	p	a	p
4H	582271120	582271417	Hvu_Mariner	p	p	p
4H	602232565	602232855	Hvu_Mariner	p	p	p
4H	602507556	602507847	Hvu_Mariner	p	p	p
4H	608761850	608762139	Hvu_Mariner	p	?	?
5H	8973267	8973559	Hvu_Mariner	p	p	p
5H	71713018	71713308	Hvu_Mariner	p	?	p
5H	126453461	126453873	Hvu_Mariner	?	?	?
5H	190672295	190676430	Hvu_Mariner	p	p	p
5H	248833829	248834120	Hvu_Mariner	?	?	?
5H	305829851	305830142	Hvu_Mariner	p	p	?
5H	345163549	345167875	Hvu_Mariner	p	p	p
5H	345168095	345169430	Hvu_Mariner	p	p	p
5H	349997599	350001680	Hvu_Mariner	p	p	?
5H	401885803	401887191	Hvu_Mariner	?	?	?
5H	437890757	437891046	Hvu_Mariner	p	a	p
5H	454200607	454200898	Hvu_Mariner	p	a	a
5H	467955146	467955517	Hvu_Mariner	p	p	p
5H	498955173	498955463	Hvu_Mariner	p	p	p
5H	540141407	540142527	Hvu_Mariner	p	p	p
5H	567589426	567589717	Hvu_Mariner	?	p	p
5H	569177072	569177553	Hvu_Mariner	p	p	?

5H	571153096	571153387	Hvu_Mariner	p	a	p
5H	581599896	581600265	Hvu_Mariner	p	?	?
5H	586791238	586791529	Hvu_Mariner	p	p	?
6H	15287510	15287877	Hvu_Mariner	p	p	p
6H	16084677	16084968	Hvu_Mariner	p	p	?
6H	22603564	22603855	Hvu_Mariner	p	p	p
6H	44348180	44348468	Hvu_Mariner	?	?	?
6H	45624645	45624992	Hvu_Mariner	p	p	p
6H	111048818	111052584	Hvu_Mariner	p	p	p
6H	151813028	151813314	Hvu_Mariner	p	p	p
6H	373490334	373490625	Hvu_Mariner	p	p	p
6H	402888693	402905335	Hvu_Mariner	?	?	?
6H	495142879	495143170	Hvu_Mariner	?	?	?
6H	514395760	514396118	Hvu_Mariner	p	p	p
6H	530593641	530593932	Hvu_Mariner	p	p	p
6H	531885773	531886073	Hvu_Mariner	p	p	p
6H	556084259	556084548	Hvu_Mariner	?	?	p
7H	6894556	6894845	Hvu_Mariner	?	?	a
7H	9490156	9490446	Hvu_Mariner	p	p	p
7H	12446521	12446811	Hvu_Mariner	?	?	?
7H	12447372	12447663	Hvu_Mariner	?	?	?
7H	17937672	17937964	Hvu_Mariner	p	p	?
7H	78561733	78562024	Hvu_Mariner	?	p	p
7H	85099722	85100013	Hvu_Mariner	p	p	p
7H	132469567	132469858	Hvu_Mariner	p	?	p
7H	164499227	164499516	Hvu_Mariner	p	p	p
7H	222796464	222796905	Hvu_Mariner	p	p	p
7H	395958786	395959078	Hvu_Mariner	?	?	?
7H	449921017	449921309	Hvu_Mariner	p	p	p
7H	451930821	451934800	Hvu_Mariner	?	?	?
7H	489997131	489997529	Hvu_Mariner	p	p	p
7H	524372772	524373062	Hvu_Mariner	p	p	p
7H	564139716	564140004	Hvu_Mariner	p	p	p
7H	586470302	586470593	Hvu_Mariner	p	?	?
7H	594138754	594144988	Hvu_Mariner	?	?	?
7H	597161082	597161372	Hvu_Mariner	?	?	?
7H	598841338	598841766	Hvu_Mariner	p	p	p
7H	601023636	601023926	Hvu_Mariner	p	p	p
7H	620445694	620445985	Hvu_Mariner	p	p	p

Table S5. The list of annotated barley genes which transcripts showed significant sequence identity to the six identified transposons

Transposons	Annotated genes	Functions	Targeted mRNA sizes (bp)	E-Values
Hvu_LINE1	HORVU.MOREX.r3.1HG0024070.1	D-3-phosphoglycerate dehydrogenase	289	1.4E-116
Hvu_LINE1	HORVU.MOREX.r3.1HG0027880.1	D-3-phosphoglycerate dehydrogenase	1258	7.56E-63
Hvu_LINE1	HORVU.MOREX.r3.1HG0032170.1	Glycosyl hydrolases family 8	307	4E-35
Hvu_LINE1	HORVU.MOREX.r3.1HG0042400.1	D-3-phosphoglycerate dehydrogenase	1169	0
Hvu_LINE1	HORVU.MOREX.r3.1HG0046810.1	D-3-phosphoglycerate dehydrogenase	630	0
Hvu_LINE1	HORVU.MOREX.r3.1HG0052910.1	D-3-phosphoglycerate dehydrogenase	950	0
Hvu_LINE1	HORVU.MOREX.r3.1HG0053080.1	D-3-phosphoglycerate dehydrogenase	1040	8E-164
Hvu_LINE1*	HORVU.MOREX.r3.1HG0056110.1	cortical cell-delineating protein-like	161	1.05E-16
Hvu_LINE1	HORVU.MOREX.r3.2HG0100140.1	D-3-phosphoglycerate dehydrogenase	1518	2E-114
Hvu_CACTA2*	HORVU.MOREX.r3.2HG0116760.1	DEAD-box ATP-dependent RNA helicase 10	51	7.17E-08
Hvu_LINE1	HORVU.MOREX.r3.2HG0135930.1	Histidyl-tRNA synthetase	296	6.63E-51
Hvu_LINE1	HORVU.MOREX.r3.2HG0178070.1	D-3-phosphoglycerate dehydrogenase	619	3.92E-60
Hvu_LINE1*	HORVU.MOREX.r3.2HG0178080.2	cortical cell-delineating protein-like	86	6.64E-13
Hvu_LINE1	HORVU.MOREX.r3.2HG0194390.1	D-3-phosphoglycerate dehydrogenase	290	2.31E-50
Hvu_LINE1	HORVU.MOREX.r3.3HG0227740.1	D-3-phosphoglycerate dehydrogenase	730	6.2E-140
Hvu_LINE1	HORVU.MOREX.r3.3HG0241840.1	D-3-phosphoglycerate dehydrogenase	420	2.03E-95
Hvu_LINE1	HORVU.MOREX.r3.3HG0252550.1	D-3-phosphoglycerate dehydrogenase	1022	1.9E-146
Hvu_LINE1	HORVU.MOREX.r3.3HG0258980.1	D-3-phosphoglycerate dehydrogenase	687	6.21E-64
Hvu_LINE1	HORVU.MOREX.r3.3HG0270850.1	D-3-phosphoglycerate dehydrogenase	813	0
Hvu_LINE1	HORVU.MOREX.r3.3HG0274120.1	D-3-phosphoglycerate dehydrogenase	1939	0
Hvu_LINE3*	HORVU.MOREX.r3.3HG0291190.1	isopentenyl phosphate kinase	77	2.06E-11
Hvu_LINE1	HORVU.MOREX.r3.4HG0351810.1	D-3-phosphoglycerate dehydrogenase	583	4.46E-91
Hvu_LINE1	HORVU.MOREX.r3.4HG0352640.1	D-3-phosphoglycerate dehydrogenase	1345	1.3E-167
Hvu_LINE2	HORVU.MOREX.r3.4HG0367520.1	Serine/threonine-protein kinase SMG1	891	0
Hvu_LINE1	HORVU.MOREX.r3.4HG0372510.1	D-3-phosphoglycerate dehydrogenase	197	8.63E-37
Hvu_LINE3	HORVU.MOREX.r3.4HG0376340.1	putative ubiquitin-like-specific protease 1B	51	3.05E-09
Hvu_CACTA2	HORVU.MOREX.r3.4HG0391140.1	chorismate synthase 2, chloroplastic	68	7.17E-08
Hvu_LINE1	HORVU.MOREX.r3.5HG0445060.1	D-3-phosphoglycerate dehydrogenase	682	0
Hvu_Gypsy1*	HORVU.MOREX.r3.5HG0465690.1	probable phytol kinase 2	87	1.43E-11
Hvu_LINE1	HORVU.MOREX.r3.5HG0470520.1	D-3-phosphoglycerate dehydrogenase	508	1.1E-111
Hvu_Mariner *	HORVU.MOREX.r3.5HG0473680.1	probable protein phosphatase 2C 68	37	3.31E-08
Hvu_LINE1	HORVU.MOREX.r3.5HG0474220.1	D-3-phosphoglycerate dehydrogenase	294	3.67E-54
Hvu_Gypsy1*	HORVU.MOREX.r3.5HG0475900.2	disease resistance protein RPM1-like	60	6.07E-10
Hvu_LINE3*	HORVU.MOREX.r3.5HG0504530.1	protein ELC-like	51	0.000000453
Hvu_Gypsy1	HORVU.MOREX.r3.5HG0514590.1	disease resistance protein RGA5-like	65	9.01E-08
Hvu_LINE1	HORVU.MOREX.r3.6HG0544570.1	D-3-phosphoglycerate dehydrogenase	963	0
Hvu_LINE2*	HORVU.MOREX.r3.6HG0582910.1	protein CbxX	48	0.000000614
Hvu_LINE1	HORVU.MOREX.r3.6HG0598480.1	D-3-phosphoglycerate dehydrogenase	1782	7E-171
Hvu_LINE1	HORVU.MOREX.r3.6HG0599980.1	D-3-phosphoglycerate dehydrogenase	618	1.28E-53
Hvu_CACTA2	HORVU.MOREX.r3.6HG0603790.1	SPX domain-containing membrane protein OsI_08463	66	3.96E-11
Hvu_LINE1	HORVU.MOREX.r3.6HG0603890.1	D-3-phosphoglycerate dehydrogenase	275	2.82E-49
Hvu_LINE1	HORVU.MOREX.r3.6HG0619970.1	D-3-phosphoglycerate dehydrogenase	419	2.2E-101
Hvu_LINE1	HORVU.MOREX.r3.7HG0657440.1	D-3-phosphoglycerate dehydrogenase	1342	0
Hvu_LINE1	HORVU.MOREX.r3.7HG0699820.1	D-3-phosphoglycerate dehydrogenase	974	8E-164
Hvu_LINE1	HORVU.MOREX.r3.7HG0700120.1	D-3-phosphoglycerate dehydrogenase	289	5.81E-58
Hvu_LINE1	HORVU.MOREX.r3.7HG0720430.1	F-box/LRR-repeat protein 13-like	77	2.32E-31
Hvu_LINE1	HORVU.MOREX.r3.7HG0684520.1	D-3-phosphoglycerate dehydrogenase	1924	0

Note: * indicated that transposons served as the UTRs of the annotated genes, other transposons served as the CDSs of the genes.

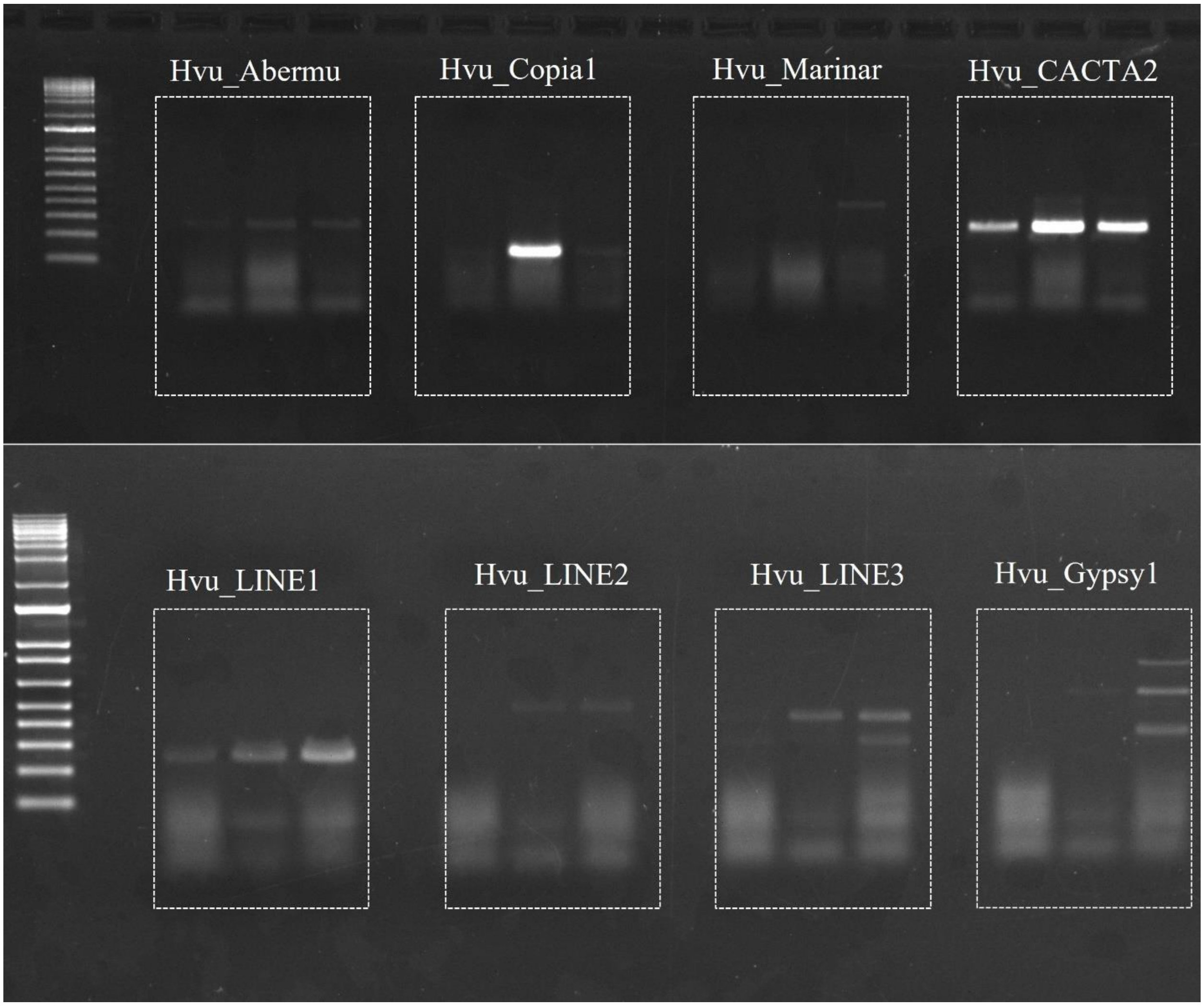

Figure S1 Full-length gel for RT-PCR analysis of eight transposons in barley. For each transposon, three types of tissues were collected, leaves, shoots and roots (from left to right).

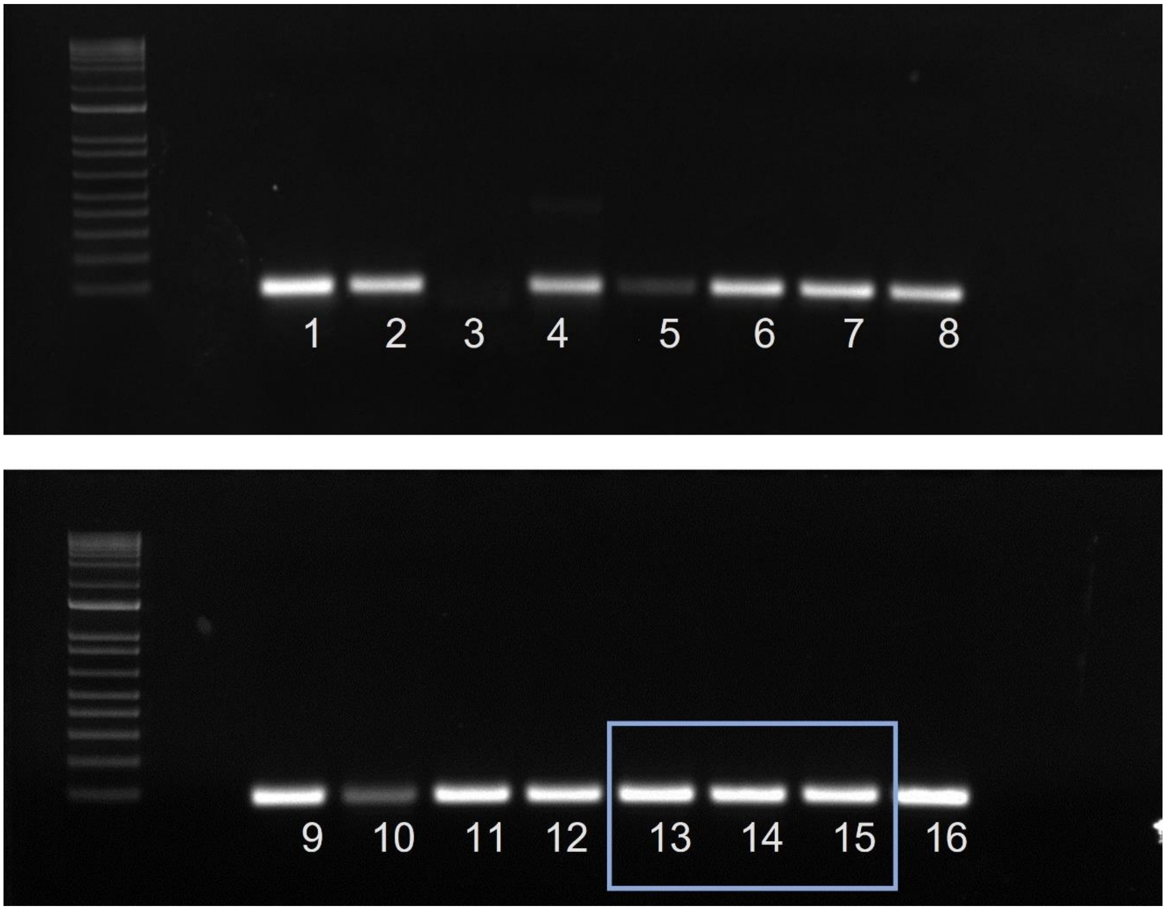

Figure S2 Full-length gel for RT-PCR analysis of actin gene in barley. The samples of 13-15 represent the cDNA from leaves, shoots and roots of 'Morex' which were then used to validate the expression of transposons.
